# Zinc deficiency as a possible risk factor for increased susceptibility and severe progression of Corona Virus Disease 19

**DOI:** 10.1017/S0007114521000738

**Published:** 2022-01-28

**Authors:** Inga Wessels, Benjamin Rolles, Alan J. Slusarenko, Lothar Rink

**Affiliations:** 1 Institute of Immunology, Faculty of Medicine, RWTH Aachen University Hospital, Pauwelsstr. 30, 52074 Aachen, Germany; 2 Department of Hematology, Oncology, Hemostaseology and Stem Cell Transplantation, Faculty of Medicine, RWTH Aachen University Hospital, Pauwelsstrasse 30, 52074 Aachen, Germany; 3 Department of Plant Physiology, RWTH Aachen University, Worringer Weg 1, 52074 Aachen, Germany

**Keywords:** Zinc deficiency, Corona Virus Disease 19, Severe Acute Respiratory Syndrome-Coronavirus-2, Impaired immune system

## Abstract

The importance of Zn for human health becomes obvious during Zn deficiency. Even mild insufficiencies of Zn cause alterations in haematopoiesis and immune functions, resulting in a proinflammatory phenotype and a disturbed redox metabolism. Although immune system malfunction has the most obvious effect, the functions of several tissue cell types are disturbed if Zn supply is limiting. Adhesion molecules and tight junction proteins decrease, while cell death increases, generating barrier dysfunction and possibly organ failure. Taken together, Zn deficiency both weakens the resistance of the human body towards pathogens and at the same time increases the danger of an overactive immune response that may cause tissue damage. The case numbers of Corona Virus Disease 19 (COVID-19) are still increasing, which is causing enormous problems for health systems and economies. There is an urgent need to reduce both the number of severe cases and the resulting deaths. While therapeutic options are still under investigation, and first vaccines have been approved, cost-effective ways to reduce the likelihood of or even prevent infection, and the transition from mild symptoms to more serious detrimental disease, are highly desirable. Nutritional supplementation might be an effective option to achieve these aims. In this review, we discuss known Zn deficiency effects in the context of an infection with Severe Acute Respiratory Syndrome-Coronavirus-2 and its currently known pathogenic mechanisms and elaborate on how severe pre-existing Zn deficiency may pre-dispose patients to a severe progression of COVID-19. First published clinical data on the association of Zn homoeostasis with COVID-19 and registered studies in progress are listed.

In March 2020, the WHO declared the Corona Virus Disease 19 (COVID-19) to be a pandemic^(1)^. Infections with Severe Acute Respiratory Syndrome-Coronavirus-2 (SARS-CoV-2) can be asymptomatic (40 % of the cases) or cause a mild illness (40 %), but in about 15 % of the cases, severe disease develops, characterised by clinical signs of pneumonia (fever, cough and dyspnoea) plus one of the following: respiratory rate > 30 breaths/min; severe respiratory distress or SpO_2_ < 90 % on room air as defined by the WHO. Patients with acute respiratory distress syndrome (ARDS), sepsis or septic shock are categorised as critically ill which is the case in about 5 % of the cases^(2)^. Amongst the co-morbidities, resulting in severe COVID-19 progression, inappropriate nutrition is increasingly attracting attention^(3)^. According to the WHO, 1·9 billion adults are overweight or obese, while 462 million are underweight^(4)^, underlining the relevance of taking inappropriate nutrition into account when discussing prevention and treatment of COVID-19. It is important to mention that Zn deficiency is frequently observed in undernutrition as well as in obesity, although the underlying mechanisms are different^(5)^.

In a previous article, we drew attention to the strong overlap of risk groups for severe progression of COVID-19 with the groups where Zn deficiency is frequently diagnosed^(6)^. The effects of Zn supplementation were described and discussed^(6,7)^. In this article, we would like to discuss how pre-existing Zn deficiency might increase the susceptibility to COVID-19 infections as well as pre-dispose individuals for severe progression of disease as summarised in Fig. 1. Despite the many improvements in Zn research, we still lack a valid biomarker to reliably assess the Zn status of an individual^(8,9)^. Serum or plasma Zn levels are often used but are not completely reliable. Thus, serum levels below 642·5 μg/l are taken as an indication of Zn deficiency, but only partially reflect intracellular concentrations and the Zn status of an individual. Therefore, clear clinical signs of Zn deficiency can be observed even if serum Zn levels are above this critical value or in the normal range. Circadian variations of serum Zn levels were observed, and serum Zn also depends upon recent food intake and the degree of hydration/dehydration of an individual^(10,11)^. Early effects of Zn deficiency are often general and include functional changes that can be associated with various diseases. For this reason, mild Zn deficiency can be ‘hidden’^(8)^. Functional deficiencies in Zn-dependent immunological processes have been shown in human subjects and mice without any significantly different serum or plasma Zn levels compared with controls^(12–14)^. Currently, Zn deficiency is mostly defined by using a combination of clinical symptoms, calculating Zn and phytate intake from food and measuring immunological changes^(15,16)^. For growing infants (<2 years) and children (<5 years), the ‘height-for-age ratio’ should be determined as an additional parameter^(17)^. Moreover, it has been suggested that serum and plasma Zn values need to be adjusted for situations where inflammation is present^(18,19)^. For these reasons, Zn deficiency is often investigated using animal models of severe and induced Zn deficiency and well-defined low-Zn diets. Alternatively, Zn deficiency can reliably be modelled in cell cultures with either Zn-depleted media or by using Zn-specific chelators. Whether the latter rather models severe or mild Zn deficiency in the context of the whole organism is hard to predict. In this review, we describe data derived from clearly Zn-deficient humans and mice. Individuals with subclinical Zn deficiency might be less severely affected, but the effects are probably still not negligible^(20)^. The consequences of Zn deficiency are manifold^(16,21–24)^, and only effects that are relevant regarding the susceptibility and progression of infectious diseases such as COVID-19 (Fig. 1) are included here. In regard to innate immunity, the article will focus on the effects of Zn on the integrity of the epithelial cell barriers, on neutrophil and macrophage maturation and functions, and in regard to adaptive immunity, we focus on lymphocyte maturation and differentiation, and cytokine and antibody production. Known effects of Zn deficiency on the vascular system and the association of those effects with diseases affecting the heart, kidney, central nervous system and intestine are described in relation to COVID-19.

**Fig. 1:**
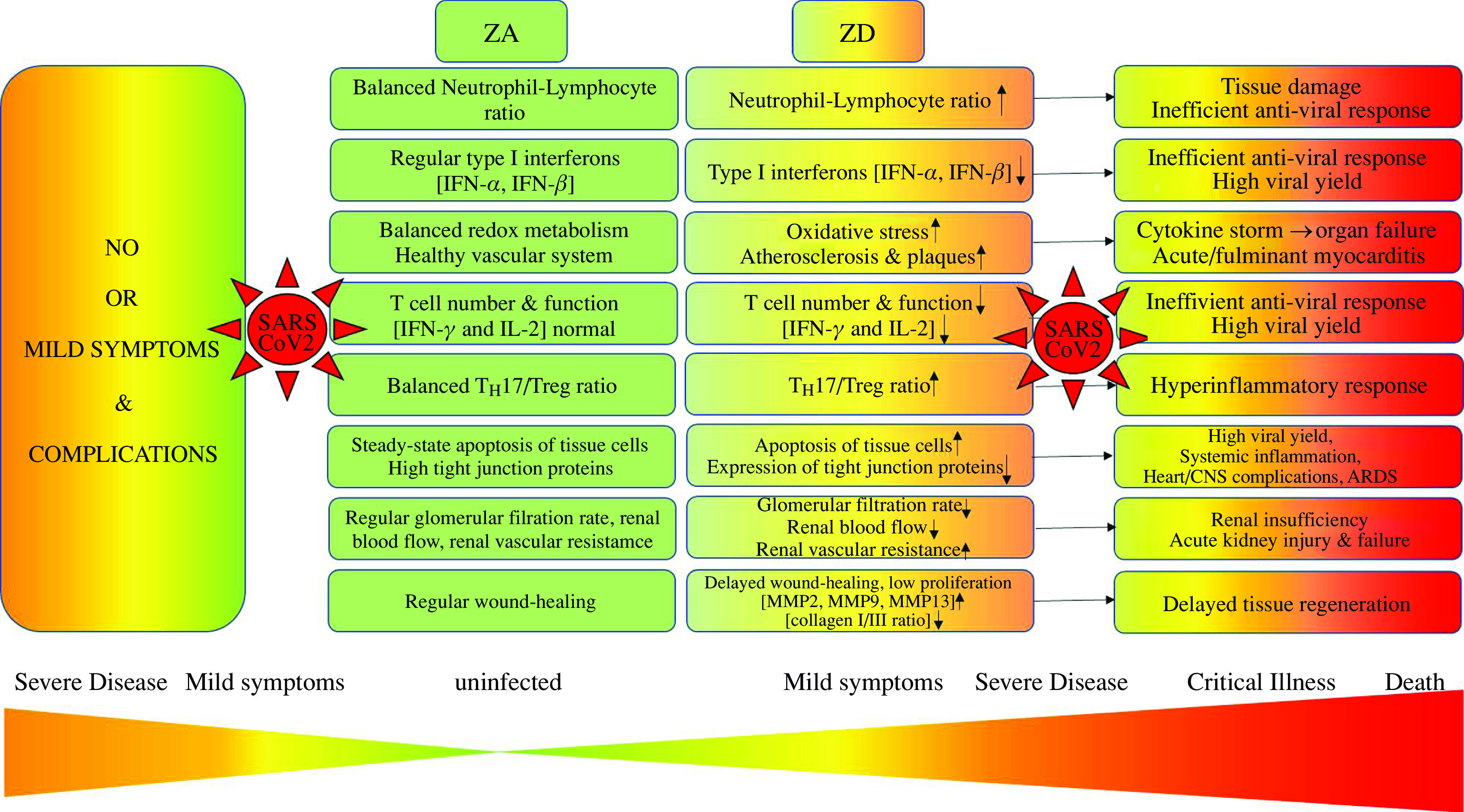
Summary of complications that can be expected in patients with pre-existing zinc deficiency, when challenged by Severe Acute Respiratory Syndrome-Coronavirus-2 (SARS-CoV-2). A patient with no co-morbidities and a balanced zinc homoeostasis will most likely develop no or mild symptoms or complications if infected with SARS-CoV-2 because immune cell numbers and functions are balanced, as are the other parameters listed in the Figure. However, zinc deficiency alone will result in the alterations indicated in the Figure. Preconditions resulting from zinc deficiency may result in the development of severe symptoms, critical illness and even death if the patient becomes infected with SARS-CoV-2. ARDS, acute respiratory distress syndrome; CNS, central nervous system; IFN, interferon; MMP, matrix metalloproteinase; T_H_, T helper cell; T_reg_, regulatory T cell; ZA, zinc adequate; ZD: zinc deficient.

In addition to nutritional causes (undernutrition, malnutrition, veganism, geophagy, a phytate-rich diet, low-Zn parenteral nutrition), conditioned Zn deficiency has been observed in association with many diseases and inflammatory reactions^(18,25)^. Attention was drawn to Zn deficiency in the 1960s due to a traditional soil-eating diet (geophagy) in a group in Iran leading to a severe Zn deficiency associated with dwarfism. The group revealed that a severely disturbed immune response, was more susceptible to infection, suffered from lethargy, and none survived beyond the age of 25 years^(16)^. Untreated severe Zn deficiency, such as that seen during *acrodermatitis enteropathica*, has a high mortality rate often because of the inefficient clearance of infections^(26)^. The subjects with *acrodermatitis enteropathica*, and the above-described group in Iran, suffered from severe Zn deficiency. However, studies in mice and human subjects have shown that detrimental effects are seen not only in severe Zn deficiency but that also a slight to moderate Zn deficiency can result in alterations of haematopoiesis and defects in the functions of immune cells^(12–14,27)^, which thus increases the susceptibility to infection. It is important to recall that the immune system is affected negatively by Zn deficiency before any other symptoms become obvious and before serum Zn levels drop below 642·5 μg/l^(28)^. Besides being essential for a robustly functioning immune system, Zn is also important for DNA synthesis, cell proliferation, cell differentiation, apoptosis, protein structure, protein–protein interactions and signal transduction as a second messenger for all kinds of cells. In the nervous system, Zn serves as an individual neurotransmitter that is secreted into the synaptic cleft^(29–33)^. Zn deficiency can manifest itself in a variety of ways; amongst others, there are increased frequencies of pneumonia and diarrhoea, an altered sense of smell and taste, cytopenia, poor wound healing, hair thinning, eczema, reduced fertility, increased fatigue, sicca syndrome and nail dysplasia^(22)^. Zn deficiency is a significant public health problem, and high numbers of deaths worldwide, especially in children, are associated with severe Zn deficiency^(8,24,34,35)^.

The magnitude of the effects of a pre-existing Zn deficiency, and the significance of mild compared with severe Zn deficiency, remains to be clearly defined and clarified in relation to COVID-19. A series of studies have been registered to analyse retrospectively the serum Zn levels of patients (online Supplementary Table S1), and the first published data in this regard are starting to appear. Data from further registered studies, investigating prophylactic Zn supplementation to decrease the susceptibility for infections and severe disease, especially in medical and military personnel, are also underway (online Supplementary Table S1). In the absence of experimental data, we extrapolate the information from the existing literature, in anticipation of the data from clinical studies, which should soon be available (online Supplementary Table S1).

## Zinc deficiency alters haematopoiesis and disturbs the balance of innate and adaptive immune cells largely to the detriment of cells from the lymphoid lineage

Severe infections with SARS-CoV-2 can cause major hematopoietic changes. Most prominently, a decrease in lymphocytes has been noted, especially affecting the T cells. In COVID-19 patients with severe symptoms, the reduction in number and the functional exhaustion of CD4^+^ as well as CD8^+^ T cells, as detected by elevated expression of Tim-3 and PD-1, is frequently described and observed early during disease^(36,37)^. The recovery of T cell numbers in severely ill patients was paralleled with the improvement of the symptoms and with positive prognosis and survival^(38)^.

Available data on the effects of SARS-CoV-2 on CD4^+^ compared with CD8^+^ T cells are somewhat controversial. While, in one study, no significant difference in the CD4^+^:CD8^+^ ratio but increased expression of CD8^+^ was found^(39)^, other studies have reported a decrease particularly of CD8^+^ T cells, or a significantly elevated CD4:CD8 ratio in COVID-19 patients^(37,38,40)^. As high levels of either perforin or granulysin, or both, were detected in CD8^+^ T cells^(41)^, it can be assumed that CD8^+^ cells are overreacting initially and are subject to exhaustion and apoptosis at later stages. However, this hypothesis remains to be addressed. In contrast, B cell numbers and serum levels of Ig (IgA, IgG and IgM) have been reported to be rather weakly affected during COVID-19^(38)^.

**Fig. 2:**
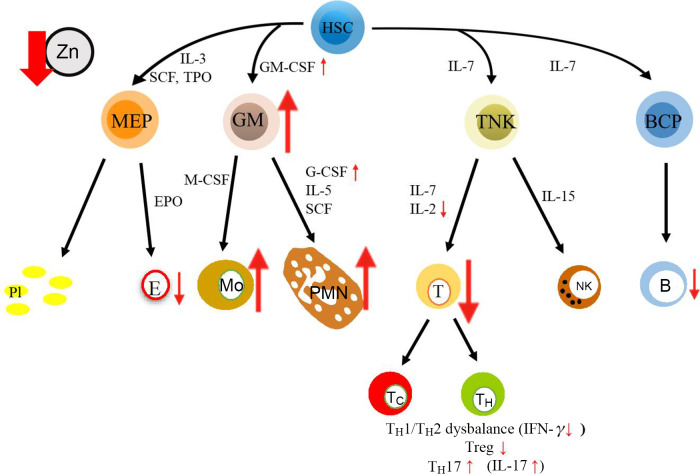
Alterations in haematopoiesis are reported during zinc deficiency as well as in Corona Virus Disease 19 (COVID-19). During zinc deficiency, indicated by the red arrow, differentiation of myeloid cells, including polymorphonuclear neutrophils (PMN) and monocytes (Mo), is prioritised over development of adaptive immune cells, this especially impacts T cells (T). Amongst others, the prioritisation of myeloid cells may be explained by changes in growth factor expression: granulocyte-macrophage colony-stimulating factor (GM-CSF) and granulocyte-colony-stimulating factor (G-CSF) were described to be highly expressed, while levels of IL-2 are decreased during zinc deficiency. Furthermore, the T helper cell (T_H_)1:T_H_2 ratio is imbalanced during zinc deficiency, Th17 cell numbers are increased, while regulatory T cell (T_reg_) numbers were described as decreased as well as their functions. Most of those haematopoietic disturbances found during zinc deficiency are generally described for COVID-19 patients, as detailed in the text. B, B cell; BCP, B-cell progenitor; E, erythrocyte; EPO, erythropoietin; GM, granulocyte-macrophage progenitor; HSC, hematopoietic stem cell; MEP, megakaryocyte–erythroid progenitor; NK, natural killer cell; Pl, platelets; SCF, stem cell factor; T_C_: cytotoxic T cell; TNK, T and NK cell progenitor; TPO, Thrombopoietin.

Haematopoiesis is severely disturbed during both severe and mild Zn deficiency, which was found in human and animal studies as illustrated in Fig. 2. Especially, a loss in pre-B cells and immature B cells, as well as early developmental T cells, including CD4/CD8 double positive and pre-T cells, was described for humans and rodents with Zn deficiency as diagnosed by low plasma Zn levels. These effects can be corrected by Zn supplementation, as shown in subjects over 65 years of age suffering from mild serum Zn deficiency and in obese subjects with decreased serum Zn levels^(5,16,27,42–44)^. Several mechanisms were described to underlie this decrease in cell numbers. Most importantly, thymus atrophy and decreased serum concentration of thymulin, which is necessary especially during maturation of T cells^(45,46)^, and lower levels of growth factors such as IL-2 (T cells) were reported in individuals with decreased serum Zn levels, and a disruption of IL-2 signalling was found when analysing cell cultures, where cellular Zn was depleted using a Zn chelator^(47)^. During dietary Zn deprivation in humans and rodents, a decreased ratio of type 1:type 2 T-helper cells, with reduced production of T-helper type 1 cytokines like interferon *γ*, is observed due to increased apoptosis^(16,30,44)^. Assuming that a Zn-deficient individual has fewer B cells compared with a person with a balanced Zn homoeostasis, a decreased generation of pathogen-specific antibodies can be expected. This might suggest that individuals, especially with a pre-existing severe Zn deficiency, would not be able to generate a sufficiently strong antibody response against SARS-CoV-2^(48,49)^.

In COVID-19 patients, especially those with severe symptoms, T_H_17 cell numbers were elevated, which is in line with the hyperinflammatory status of the immune system^(41)^. Recent studies underline that differentiation into the main CD4^+^ cell subtypes is disturbed when Zn supply is low. *In vivo* data from patients with allergic asthma reveal that impaired T_reg_-mediated suppression can be correlated with decreased serum Zn levels^(50)^. Data from *in vitro* differentiation experiments, using Zn-deficient compared with Zn-adequate culture medium, further strengthen the hypothesis, that development of proinflammatory T_H_17 cells is supported as a consequence of Zn deficiency^(51)^.

In contrast to the consistently observed lymphopenia in COVID-19 patients, the numbers of myeloid cells in the blood and in lung tissue were strongly elevated. Neutrophilia was associated with the progression of severe disease to ARDS and with increased mortality therefrom, similarly to that described for bacteria-induced lung injury^(52–55)^. As blood analyses of non-survivors revealed severe lymphopenia combined with significantly elevated numbers of neutrophils, the neutrophil:lymphocyte ratio was suggested as a prognostic marker for COVID-19 patients^(38,55)^. Similar to the neutrophil:lymphocyte ratio shift in COVID-19 patients, the balance between adaptive and innate immune cells is shifted towards the latter during Zn deficiency. Investigation of severely Zn-deficient rodents, that were fed on a low-Zn diet, showed high numbers of neutrophils and their specific products in the bone marrow and blood compared with Zn-adequate animals^(49,56,57)^. Our own unpublished results suggest that maturation of myeloid precursors into granulocytes in Zn-deficient human cell cultures is also increased compared with cells that differentiate in Zn-adequate cell cultures, while Zn supplementation attenuates the development into mature neutrophils^(58)^. As an underlying mechanism, the increased response to growth factors, for example, to the granulocyte-macrophage colony-stimulating factor and granulocyte-colony-stimulating factor, can be named, as was determined in cell culture experiments where Zn was added to the culture medium^(54,59)^.

Thrombocytopenia, which we will come back to later, and a decline in Hb are also common in COVID-19 patients^(37)^. Lower Hb and erythrocyte counts were found in severe COVID-19 cases compared with moderate cases. Furthermore, higher ferritin was found in severe COVID-19 cases, and a significant difference in the mean ferritin levels was found between survivors and non-survivors^(60)^. Additional research is necessary to prove the suggested important role of anaemia and a disturbed Fe in severe cases of COVID-19. This might uncover new treatment options.

Alterations in bone marrow metabolism were related to decreased serum Zn concentrations in humans^(21,61)^. This finding together with the observation that the osmotic fragility of erythrocyte membranes is elevated in animals with dietary Zn deficiency, as are levels of lipid peroxidation in mitochondrial and microsomal membranes, suggests that there might be some interconnection between Zn deficiency and anaemia^(62)^. Indeed, serum hypozincaemia is commonly observed in anaemic subjects^(61,63–66)^. However, importantly, a causal association between Zn deficiency and anaemia has so far not been established clearly and is discussed controversially. For example, serum Zn concentration was correlated with serum ferritin concentration in patients undergoing peritoneal dialysis^(67)^. Morover, lower serum ferritin was significantly correlated with smaller sizes of Zn pools in premenopausal women, although without anaemia^(68)^. Regarding the effects of adjuvant Zn therapy for improving anaemia in haemodialysis patients, Hb levels were found to increase significantly in Zn-supplemented patients compared with patients not supplemented with Zn. The authors suggest a ‘zinc deficiency anemia’, which needs further evaluation^(64)^. In this regard, it should be pointed out that nutritional deficits often include several elements concomitantly, as shown for Zn and Fe, Se and others^(10,63,65,69)^. Since the association of anaemia with an increased risk and severe progression of COVID-19 has not been clearly established, this will not be discussed further in this article. However, as anaemia is generally related to poor outcomes of infectious diseases^(70)^, possible nutritional deficits in COVID-19 risk groups should be addressed and not only Zn but also Fe, Se and other elements might need to be supplemented if applicable.

Comparing the disturbance of haematopoiesis observed in individuals with low serum Zn levels or with COVID-19, various congruencies become apparent. As lymphopenia, neutrophilia and a decline in Hb are associated with progression to severe COVID-19, it can be hypothesised that a pre-existing severe Zn deficiency will predispose patients to stronger progression of infections with SARS-CoV-2 and that even a mild Zn deficiency should be corrected to prevent more severe progression of the viral infection. A pre-existing elevated neutrophil:lymphocyte ratio, even one of low magnitude, as during Zn deficiency, might be detrimental in the case of severe and aggressive infections such as COVID-19. At first sight, elevated numbers of innate immune cells as a first line of defence might appear beneficial. However, they are easily overrun during viral infections as a specific response, and the release of anti-viral factors and antibodies, especially by adaptive immune cells, is of major importance here. The elevated numbers of hyperactivated innate immune cells can even lead to high levels of inflammatory factors and oxidative stress causing destruction of host tissue. In SARS-CoV-infected mice, the recruitment of high numbers of monocytes and macrophages to the lungs was observed, secreting high numbers of proinflammatory cytokines and chemokines, which are associated with vascular leakage, underlining the detrimental effect of highly reactive immune cells^(71)^. Similar scenarios are suggested for SARS-CoV-2 in humans^(72–74)^.

The next chapters will show that the alterations in immune cell counts are not the only indication of an association between Zn deficiency and COVID-19.

## Pre-existing zinc deficiency could prime for the cytokine release syndrome

A frequent complication among patients with severe COVID-19 is the cytokine release syndrome, which spreads throughout the body from the focal infected area and may lead to death because of subsequent ARDS or multiple organ dysfunction syndrome and other complications^(75,76)^. It was reported that among the COVID-19 patients, the classic serum proinflammatory cytokines like TNF-*α*, IL-2, IL-6, IL-7, IL-8, IL-10, granulocyte-colony-stimulating factor and C-reactive protein are elevated^(72,73,76)^. IL-6 in particular, which is produced by lung resident macrophages and circulating immune cells^(77–79)^, has been associated with severe COVID-19 and increased mortality^(80)^. Moreover, D-dimers, ferritin, lactate dehydrogenase, aspartate aminotransferase, alanine aminotransferase and soluble CD25 (IL-2 receptor) are increased, while fibrinogen is decreased^(72)^.

Although there are planned and ongoing trials to counter the cytokine storm using approved antibodies such as tocilizumab (anti-IL-6 receptor), anakinra (IL-1 receptor antagonist, IL-1RA) and anti-TNF antibodies used to treat other hyperinflammatory conditions, and in spite of some benefits, so far their efficacy was not proven in large-scale, randomised controlled trials^(72,81–83)^, and therefore, therapeutic options are still limited.

In recent years, *in vivo* and *in vitro* data supporting the hypothesis that a pre-existing Zn deficiency augments the activation-induced inflammatory response, and results characterizing the possible underlying mechanisms, are constantly accumulating. Serum hypozincaemia was correlated with increased serum levels of, amongst others, IL-1*β*, IL-6, TNF*α*, IL-8, granulocyte-colony-stimulating factor, IL-10, IL-1RA, IL-17, C-reactive protein and calprotectin; thus, a whole battery of proinflammatory mediators is increased, especially during severe Zn deficiency, and particularly in combination with the inflammatory response to a pathogen^(54,84–88)^. In the case of IL-1*β* and TNF*α*, Zn chelation was shown to induce epigenetic changes in the promoters of both genes in cell culture experiments. More specifically, the accessibility of regions in the DNA close to the transcriptional start site was significantly increased so that after inflammatory activation of the cells, by, for example, lipopolysaccharide, gene expression was augmented^(88)^. Moreover, activation of NF*κ*B a central player in the signalling pathways involved in the generation of inflammatory factors is increased when Zn is limiting, as found in mice with diet-induced serum hypozincaemia^(87)^. Cell culture experiments using Zn-depleted medium revealed increased expression of calprotectin in myeloid precursors and mature monocytic cells^(89)^

Activated T cells express lower IL-2 and interferon *γ* mRNA levels, as was shown *in vitro* and observed in individuals with low serum Zn^(90)^. IL-2 is essential for natural killer cell and cytotoxic T lymphocyte activity. Interferon *γ* is essential for killing viruses, parasites and bacteria. Thus, the decreased efficiency of the immune response in Zn-deficient subjects is easily explained^(14,90,91)^. Defects in T cell function as a consequence of Zn deficiency can also be explained by the accumulation of deoxyguanosine, which results from decreased Zn-dependent nucleoside phosphorylase activity in human lymphocytes, derived from human Zn-deficient volunteers before and after Zn supplementation^(92)^. Serum Zn deficiency strongly affects Th1 cells, while Th2 cells are largely unaffected, and production of IL-4, IL-6 and IL-10 (Th2 cytokines) remains rather stable. However, production of interferon *γ* and IL-2 (Th1 cytokine) is decreased^(93)^.

Although Treg cell numbers might be constant, or even elevated, during *in vitro* differentiation under Zn deficiency, it was suggested that their function is disturbed^(51)^. *In vivo* data are so far scarce, but some studies in mice suggest decreased transforming growth factor *β* (Treg cytokine) levels during Zn deficiency, pointing to a malfunctioning of Treg cells and thus imbalance of the immune response^(94)^. As Treg cells are important master regulators within the immune system, essential for tolerance and balance and differentiation of the remaining CD4^+^ T cell subtypes, a disturbed immune response can be expected.

Treatment of cell cultures with a Zn chelator disturbed the cytotoxic activity of natural killer cells^(95,96)^. Similar effects were reported for rats fed on a Zn-deficient diet^(97)^. This effect might decrease the killing of host cells which become infected by the virus.

A number of effects described above are due to the requirement of Zn for intracellular signal transduction and the consequent disruption of a multitude of signalling pathways when Zn supply is limited. Zn’s effect on phosphatases and kinases is central here, as is its ability to induce changes in membrane fluidity and thus receptor expression and dimerization as found *in vitro* and *in vivo* ^(47,59,98)^. Finally, epigenetic changes occur during Zn deficiency as described above as found in various models of Zn deficiency^(99,100)^.

In the case of IL-6, another connection to Zn deficiency has been described. A SNP was found in the IL-6 gene at position −174. It is associated with a disturbed age-related Zn deficiency, and it seems to be relevant during the regulation of Zn-related genes such as metallothioneins. The frequency of this polymorphism increases with age and offers an additional explanation for the high risk of Zn deficiency described for the elderly^(46,101)^. Interestingly, the IL-6–174 SNP was also associated with an increased risk for severe progression of and mortality from COVID-19, as was suggested previously for sepsis, but never proven up to now^(102–104)^. Individuals with this SNP could be actively supplemented with Zn, not only to help prevent severe COVID-19 but also to enable a balanced immune response in general^(46,105)^.

Glucocorticoids were suggested as a means to attenuate the cytokine storm and proposed as a treatment option during the hyperinflammatory phase of COVID-19^(72)^. On the other hand, chronically increased glucocorticoids may augment lymphopenia^(106,107)^. Serum Zn deficiency was associated with chronically elevated levels of glucocorticoids, especially corticosteroids. However, data are not clear in this regard yet, and studies have been published not recommending the use of glucocorticoids during COVID-19 treatment, or at least recommend caution. Criticism of glucocorticoid use is largely based on data on SARS from 2003, where improper use of systemic corticosteroids increased the risk of osteonecrosis of the femoral head, which is, however, a classical side effect of glucocorticoid therapy and not related to the virus^(108–111)^. At first sight, this is one of the only consequences of Zn deficiency that might be viewed as an advantage in terms of COVID-19. In this regard, it should also be mentioned that the chronically increased glucocorticoid levels were suggested to be associated with the increased apoptosis of lymphocytes and probably also of cells of the thymus, thus explaining thymic atrophy in mice and humans with decreased serum Zn levels^(112–114)^. However, those suggestions require experimental verification.

The cytokine storm is central to the progression from mild or severe disease to complications and critical illness associated with COVID-19 and should be prevented by any possible means. The hyper-inflammation is largely involved in damaging various organs, including the lung, heart, liver, kidney and probably also the intestine and the brain. Interestingly, the central nervous system, the gastrointestinal tract, lungs, liver, the epidermal, reproductive and skeletal system are clinically affected by severe Zn deficiency which causes elevation of inflammatory markers^(16,62)^. As the treatment of the cytokine storm is complex and the individual patient response to certain treatments is almost impossible to predict, the best option is to prevent the cytokine storm. Thus, groups that are at risk of Zn deficiency should be supplemented routinely. Of course, individuals with severe pre-existing Zn deficiency will benefit the most; however, adjusting mild Zn deficiencies is also of importance especially in individuals from COVID-19 risk groups such as the elderly, diabetic patients and individuals with heart and vascular co-morbidities.

Additional roles of Zn in the regulation of immune cell function, but perhaps not obviously relevant for what is known of SARS-CoV-2 infection, have been reviewed extensively elsewhere^(25,29,44,51,87,96,99,115,116)^.

## Zinc deficiency and vascular complications: possible association with complications affecting multiple organs

Cardiovascular complications are frequently reported during COVID-19, especially in patients with pre-existing pathologies of the heart and vascular system, such as atherosclerosis^(79,117)^. Venous, arterial and microvascular thromboses are increased in patients with COVID-19. Moreover, COVID-19-associated hypercoagulopathy closely resembles the pathophysiology and phenotype of complement-mediated thrombotic microangiopathy^(117)^. An increase of proinflammatory cytokines, increased complement activation, endothelial dysfunction and immunothrombosis are considered to be key mechanisms of hypercoagulopathy. For instance, venous thromboembolisms, also driven by a hyperinflammatory milieu, were described in 20–31 % of severe COVID-19 cases^(37,118–121)^. Moreover, an increased number of especially polymorphonuclear neutrophils (PMN) together with high amounts of neutrophil extracellular traps were observed in the thrombi of COVID-19 patients^(122)^. Arterial embolism, including acute pulmonary embolism, ischaemic stroke and acute myocardial injury, was also increased in patients with severe SARS-CoV-2 infection^(123–126)^. Subsequent thrombocytopenia was associated with poor prognosis for COVID-19 patients. Concerning the endothelial dysfunction, it was proposed that direct endothelial damage can lead to an increased thrombogenic effect in the microcirculation^(127)^. An impaired microcirculation can cause complications in various organs including the lung, the kidneys, the heart, the brain, the liver or the pancreas.

As already indicated, an increased activation and tissue recruitment of PMN in Zn-deficient individuals are likely^(54,128,129)^. Thus, pre-existing Zn deficiency may be indirectly associated with thrombus formation. The association of pre-existing Zn deficiency with hyperinflammation was already described in this article and can also be related to an increased risk for thromboembolism. In addition, Zn is essential for various aspects of physiological coagulation and might impact thrombogenesis as well as fibrinolysis^(130,131)^. However, Zn’s effects seem to depend on the microenvironment and might be locally restricted and temporary^(130)^. For example, Zn can be secreted by activated platelets resulting in locally increased Zn concentrations in the vicinity of a thrombus, while the systemic Zn homoeostasis remains probably rather stable. The direct effects of pre-existing Zn deficiency on coagulation are not entirely clear. Studies in Zn-deficient humans, rodents and guinea pigs revealed clotting abnormalities, impaired platelet function as well as an increased and prolonged bleeding tendency^(132,133)^. Those Zn-deficiency-induced defects were reversible by Zn supplementation^(134)^. In a recent *in vitro* study, Zn deficiency inhibited the agonist-activated production of reactive oxygen species (ROS) by platelets and decreased glutathione levels and glutathione peroxidase activity, which might result in altered thrombus formation^(135)^. Due to the lack of detailed and consitent data, a clear conclusion on the effects of Zn deficiency regarding fibrinolysis and coagulation cannot be drawn. However, as with many topics discussed in this review, a well-balanced Zn homoeostasis seems to be key to a physiological balance also in the example of coagulation and fibrinolysis.

The risk of developing an acute coronary syndrome during SARS-CoV-2 infection is especially increased in patients with atherosclerotic vascular disease^(136)^. The development and subsequent rupture of vulnerable plaques can result in heart attack or stroke, and subsequent heart failure and death^(137,138)^. During the development of atherosclerosis, up-regulation of adhesion molecules on endothelial cells is one of the central events, largely involving the transcription factor NFκB. An increased activation and DNA binding of selected transcription factors during Zn deficiency were established *in vitro* ^(139,140)^. In addition, the role of Zn in NFκB-related signalling has been described in various studies^(87,96)^. The association of severe pre-existing serum Zn deficiency in mice with an increased risk of atherosclerosis was additionally explained by the Zn-dependent alteration of endothelial surface markers, changes of the plasma lipid composition and the promotion of the proinflammatory milieu^(85,96,141)^. Results from various *in vivo* and *in vitro* studies as summarised by Choi *et al.* indicate that Zn supplementation may reduce the risk of atherosclerosis and protect against myocardial infarction as well as ischaemia/reperfusion injury^(23)^. The vasculitis described in COVID-19 patients resembles the reaction to infections with *Varicella zoster* virus, where the viral replication in the cerebral arterial wall directly triggered local inflammation^(142)^. Zn supplementation in cell culture experiments was shown to decrease viral replication^(143)^, and Zn supplementation might thus attenuate virus-induced vasculitis. Recently, a molecular modelling study predicted an interaction of Zn with RNA-dependent, RNA-polymerase and 3C-like proteinase enzymes of SARS-CoV-2, which awaits experimental verification.

Associations of diseases such as arterial hypertension, atherosclerosis, congestive heart failure and CHD are described in both Zn deficiency and COVID-19^(85,144–150)^, but a causal link between Zn deficiency and the observations in COVID-19 remains to be established.

## Pre-existing zinc deficiency is associated with severe progression of respiratory diseases

SARS-CoV-2 enters the human body predominantly via the respiratory tract. In healthy individuals, viral entry is hampered by the mucous-coated membrane of the alveoli as well as the immune cells and their anti-viral products protecting the lungs^(151)^. When SARS-CoV-2 has crossed the epithelial barrier, it can elicit extensive alveolar injury and pulmonary fibrosis, which are irreversible pathological changes. The progression of mild COVID-19 to pneumonia, acute lung injury and subsequently to ARDS is the leading cause of mortality, affecting 5–10 % of the COVID-19 patients worldwide^(152,153)^.

**Fig. 3:**
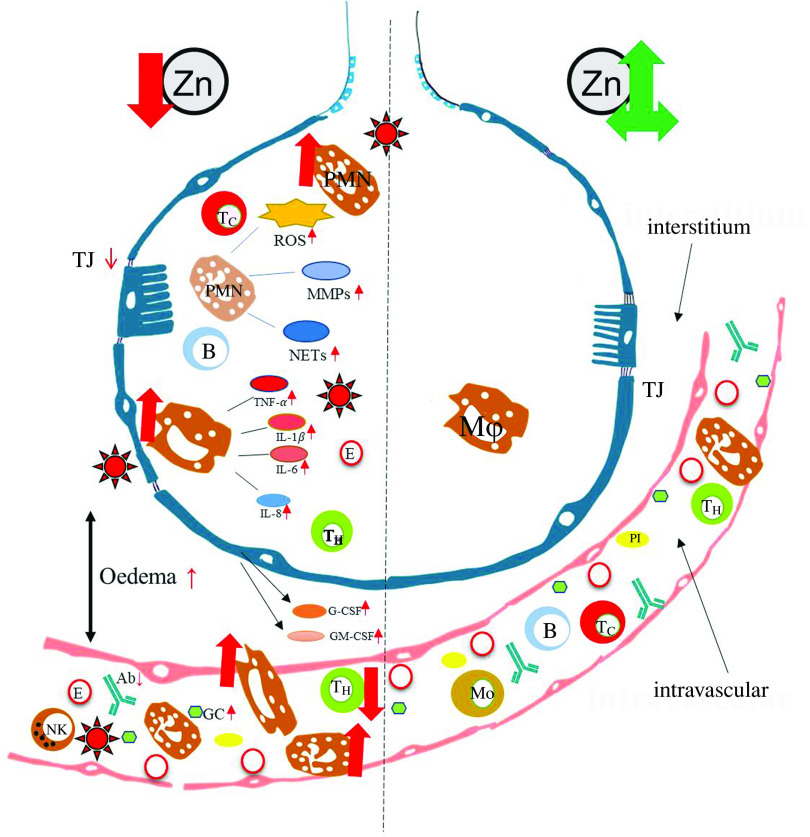
Pulmonary effects observed in Severe Acute Respiratory Syndrome-Coronavirus-2 (SARS-CoV-2) infected patients with pre-existing zinc deficiency as compared with patients with a balanced zinc homoeostasis. Pre-existing zinc deficiency (left) was suggested to increase the number, recruitment and inflammatory potential of especially PMN to the insides of the bronchi. Lymphocyte numbers are generally decreased, most prominently affecting T helper cell (T_H_) cells. The zinc deficiency-related decrease in tight junction expression and the increase in endothelial cell apoptosis have several consequences. Thus, infiltration of the lung by host cells, as well as the leakage of pathogens such as SARS-CoV-2 and secondary pathogens such as *Streptococcus pneumoniae* into the vascular system, is frequently observed during zinc deficiency. Detailed explanations can be found in the text. For comparison, the characteristics of zinc-adequate individual are indicated on the right. Ab, antibody; B, B cell; E, erythrocyte; G-CSF, granulocyte colony-stimulating factor; GC, glucocorticoid; GM-CSF, granulocyte-macrophage CSF; MMP, matrix metalloproteinase; Mo, monocyte; Mϕ, macrophage; NET, neutrophil extracellular trap; NK, natural killer cell; Pl, platelet; PMN, polymorphonuclear neutrophil; ROS, reactive oxygen species; Tc, cytotoxic T cell; TJ, tight junction; ZA, zinc adequate; ZD, zinc deficient.

As illustrated in Fig. 3, the expression of tight-junction proteins is decreased under Zn-deficient conditions. This as well as reduced expression of adherens junction proteins reduces the integrity of the endothelial barrier and might facilitate viral entry, as shown in a variety of studies investigating human and rodent tissue *in vivo, ex vivo* and *in vitro* ^(54,141,154–157)^. Experiments using an *ex vivo* model of differentiated human airway epithelium showed that exposure to Zn-depleted medium significantly augmented the down-regulation of the tight junction proteins such as Zonula Occludens-1 and Claudin-1 that was induced by cigarette smoke extract^(154)^. Another study, which investigated primary human upper airway and type I/II alveolar epithelial cells that were grown in Zn-depleted compared with Zn-adequate medium, revealed that Zn deprivation augmented activation-induced proteolysis of E-cadherin and *β*-catenin, both adherens junction proteins^(157)^. Since intracellular Zn levels of endothelial cells largely depend on the protein-bound Zn pool in the blood serum, the cells are deprived of Zn during serum hypozincaemia. Low endothelial Zn disturbs cellular metabolism and is associated with oxidative stress. Increased serum levels of oxidised LDL and high amounts of inflammatory cytokines derived from activated monocytes are frequently observed in individuals with serum Zn deficiency, and together with the high oxidative stress, this leads to increased apoptosis of epithelial cells. Consequently, mild pre-existing Zn deficiency combined with inflammation-induced serum hypozincaemia may exacerbate epithelial barrier permeability of the lung in COVID-19 patients.

Previous investigations on SARS-CoV-1 infections revealed that phagocytic cells largely contributed to the antibody-mediated elimination of the virus^(158)^. Amongst the phagocytes, resident macrophages are constantly patrolling the lung, while high numbers of PMN are recruited during infections, abundant ones. PMN are highly reactive cells, equipped with their complete anti-microbial weaponry when they leave the bone marrow. Upon activation, they release their granular content which includes highly reactive mediators such as ROS, reactive nitrogen species, antimicrobial peptides, matrix metalloproteases that degrade extracellular matrix and more^(159,160)^. Those factors are primarily secreted to destroy invading pathogens. However, if secreted in excessively high amounts, they can destroy the host tissue as well^(161)^, as was suggested to explain tissue injury in SARS-CoV-2 infections.

**Fig. 4:**
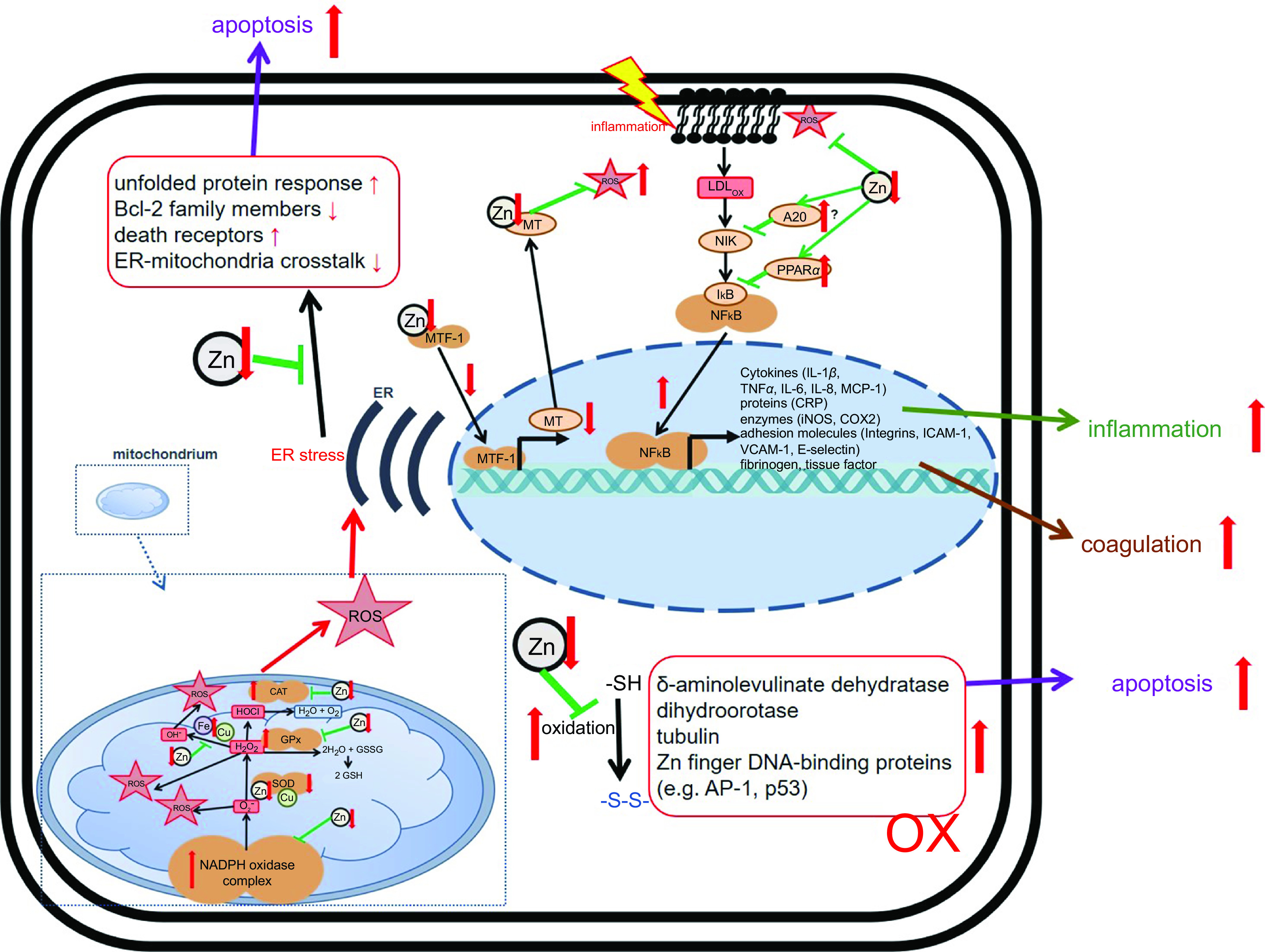
Effects of zinc deficiency on stress-induced changes in redox metabolism. Green arrows indicate zinc-dependent cellular functions. Red arrows illustrate the effects of zinc deficiency. A detailed description of the mechanisms underlying disturbed redox metabolism during zinc deficiency can be found in the text. AP-1, Activator protein 1; Bcl-2, B-cell lymphoma 2; CAT, catalase; COX, Cyclo-oxygenase; CRP, C-reactive protein; ER, endoplasmic reticulum; GPx, glutathione peroxidase; ICAM, intercellular adhesion molecule-1; iNOS, inducible nitric oxide synthase; MT, metallothionein; MTF, metal-responsive transcription factor-1; Ox, oxidated; MCP, monocyte chemoattractant protein; NIK, NFκB-Inducing Kinase; ROS, reactive oxygen species; SOD, superoxide dismutase; VCAM, vascular cell adhesion molecule.

With respect to PMN activity, the effects of Zn deficiency are not clearly defined. While some studies describe attenuated motility of PMN in moderately Zn-deficient individuals^(162,163)^, the numbers of PMN found in the infected tissues of animals with pre-existing Zn deficiency are higher compared with animals with adequate Zn supply^(128,129)^. Whether the defect in chemotaxis is compensated by the elevated numbers of PMN observed in Zn-deficient rodents, remains to be investigated^(49)^. The formation of ROS and neutrophil extracellular traps by PMN was reported to be decreased in Zn-deficient cells in culture^(164)^. Surprisingly, Zn supplementation of mice *in vivo,* or of human neutrophils in cell culture, also decreased activation-induced neutrophil extracellular trap formation^(54)^. In this context, we would like to mention that it was shown for various cell types, mostly in cell culture models, that Zn deficiency alters redox metabolism and results in oxidative stress^(165–169)^. There are several suggestions for the mechanisms responsible for the elevated ROS levels in Zn-deficient conditions, as summarised in Fig. 4. First, Zn deficiency was related to the decreased activity of enzymes which are central to ROS metabolism, such as the Cu/Zn superoxide dismutase *in vitro*. Here, the inactivation of enzymes due to the lack of Zn in their catalytic centre was described^(168,170,171)^. Second, expression of metallothioneins, not only the major intracellular Zn binding proteins but also an important free radical scavenger, is decreased during Zn deficiency, which was shown using various models of Zn deficiency and was recently summarised^(168)^. As a third mechanism, Zn is necessary to protect the free sulfhydryl groups in proteins from oxidation. A lack of Zn might also alter the formation of intramolecular disulphide bonds, causing steric hindrance and conformational changes, which can be associated with increased activity or the loss of function of molecules involved in balancing the redox state of the cells, determined in cell culture experiments and suggested by *in vivo* examination of Zn-deficient animals^(172)^. In Zn-adequate conditions, Zn competes with other redox-active metal ions with similar coordination chemistry such as Cu or Fe for protein binding. The lack of Zn as competitor is a fourth suggested mechanism explaining the increased oxidative stress when Zn is limited. This was investigated for the oxidation of myoglobin and the activity of superoxide dismutase^(170,173)^. Zn also competes with Fe and Cu for binding to the NADPH oxidase and usually inhibits NADPH oxidase activity. Increased NADPH oxidase activity was reported for neuronal cells cultured in the Zn-depleted medium^(174)^. In this context, Zn can bind NADPH, but not NADH, and thus inhibits NADPH-dependent enzymes *in vitro* ^(175,176)^. Moreover, Zn interferes with the Fenton reaction *in vitro* suppressing lipid peroxidation^(177,178)^. As a fifth point, Zn deprivation was associated with dysfunctions of mitochondria and the endoplasmic reticulum. Finally, Zn’s effect on gene expression might affect redox metabolism. Zn was shown to be involved in the up-regulation of several transcription factors, and some antioxidant molecules such as glutathione and detoxifying enzymes such as glutathione S-transferase and haemeoxygenase-1 mostly investigated using Zn-deficient cell cultures^(176,179)^. The nuclear factor erythroid 2-related factor 2 can be induced by Zn, as was investigated in rats fed on a low-Zn, Zn-adequate or high-Zn diet^(176,179,180)^. Whether Zn deficiency has the opposite effect to Zn supplementation remains to be explored, but in summary, the multiple mechanisms described above can explain the overall increase in ROS during Zn deficiency, which was consistently found in various models of Zn deficiency. We thus hypothesise that in combination with the infection-induced inflammation observed in COVID-19 patients, pre-existing Zn deficiency might augment the formation of ROS and reactive nitrogen species causing severe tissue damage. On the other hand, the anti-oxidative properties of Zn are widely described and accepted^(23,140,181,182)^, suggesting benefits of Zn supplementation for COVID-19 patients.

COVID-19 often shows systemic effects in the patient’s tissues and organs, often resulting in multi-organ failure and high death rates^(3,74,82,183)^. Furthermore, ‘septic shock’ is another cause of mortality from SARS-CoV-2 and is currently observed in 4–8 % of COVID-19 patients^(52,53,184)^. When reading all the articles on COVID-19 discussing the symptoms in individuals undergoing mild compared with severe viral disease, one cannot help but notice the parallels to mild bacterial infections compared with bacterial sepsis and its progression to ARDS^(37,161,185)^. Regarding bacterial sepsis, various studies in animals and humans describe an association of disease progression and mortality in relation to the Zn status, which could be extrapolated to COVID-19. It was shown that pre-existing Zn deficiency was a prerequisite for the progression from mild inflammation to pneumonia and severe sepsis in mice. Severity of disease was monitored by analysing the serum levels of proinflammatory cytokines (i.e. assessment of the cytokine storm) and damage to the lungs, the liver and the kidney. Also, serum Zn concentrations were inversely correlated with sepsis severity. Thus, serum Zn was suggested as a prognostic marker for mortality in septic mice, pigs, adult humans and infants. In critically ill children, complications of sepsis, the necessity for mechanical ventilation and resulting mortality rates were correlated with low serum Zn levels.^(101,128,129,186–193)^. Moreover, Boudreault *et al.* revealed that pre-existing Zn deficiency primes the lungs for severe complications derived from mechanical ventilation, including the progression from acute lung injury to ARDS^(194)^. In cystic fibrosis, Zn deficiency, caused by a splice switch in the Zn Importer ZIP2, caused hypersecretion of the glycoprotein mucin in airway epithelial cells, significantly increasing disease severity^(195)^. Pre-existing serum Zn deficiency was implicated to be responsible for the high incidence of pneumonia in elderly, hospitalised patients^(192,196,197)^. Enhanced infection and virulence of *Streptococcus pneumoniae* in Zn-deficient mice were reported. In addition to disrupted epithelial barriers and inadequate immune response, the enhanced virulence was explained by the sensitivity of *S. pneumonia* to Zn intoxication, reduced during Zn deficiency^(189)^. Direct effects of Zn deficiency on viral replication have not been addressed to date. Finally, the correlation between Zn deficiency and infection severity may be due to reverse causality, that is, the negative effects that inflammation has on serum Zn concentration. We thus suggest that when the serum Zn levels fall below a certain threshold, the inflammatory response will be self-perpetuating. Again, most tissue damage and detrimental consequences can be expected for patients with pre-existing severe Zn deficiency, but in view of the manifold effects of already mild deficiency, normalising the Zn status offers an easy and cost-efficient approach to reduce disease symptoms.

The hypothesis that Zn deficiency is a risk factor for severe COVID-19 progression and the development of pneumonia and ARDS is supported by successful supplementation studies using Zn to prevent or attenuate respiratory diseases, as we summarised previously^(6)^. Moreover, first data indicating the congruency of low-Zn status of COVID-19 patients as well as the inverse correlation between serum Zn levels and COVID-19 severity were recently published^(10,198)^. However, the low serum Zn levels might, once more, be the result of the severe inflammatory response elicited by the virus^(18)^. Clear data on possible pre-existing serum Zn defiicencies are still lacking.

## Disrupted epithelial barrier integrity during zinc deficiency: opening the way for Severe Acute Respiratory Syndrome-Coronavirus-2 and co-infections

Evidence is accumulating that, in addition to attacking the lungs and the respiratory tract, SARS-CoV-2 frequently damages other organs (heart, vessels, nerves/brain, kidneys and skin). Disruption of tissue barriers is an integral part of the pathophysiology of infectious diseases, as it facilitates distribution of the pathogen within the body^(151)^. The effects of Zn deficiency, described above regarding the lung endothelial barrier, were similarly described for other endothelial layers, including those of kidney, liver, intestine and brain.

It should also be mentioned that the expression of ACE2, lately also called ‘SARS-CoV-2 receptor’, is not limited to the lungs, that is, the goblet and ciliated epithelial cells of the upper airways, alveolar (Type II) epithelial cells and cells of the pulmonary vasculature. ACE2 is also expressed on migratory angiogenic cells, and vascular smooth muscle cells, cardiofibroblasts, cardiomyocytes, pericytes and epicardial adipose cells of the heart; glomerular endothelial cells, podocytes and proximal tubule epithelial cells of the kidneys; cholangiocytes and hepatocytes of the liver; pigmented epithelial cells, rod and cone photoreceptor cells and Müller glial cells of the retina; enterocytes of the intestines and on cells from circumventricular organs of the central nervous system. Binding of SARS-CoV-2 was claimed to result in the loss of ACE2 from the cell surface due to receptor endocytosis and proteolytic cleavage^(199)^. On the other hand, ADAM17-mediated ACE2 shedding facilitates SARS-CoV-1 entry and induces tissue damage by TNF-*α* production, which remains to be shown for SARS-CoV-2^(200)^. However, disturbed ACE2 expression levels on the cell surface and increased viral entry result from both scenarios. Amongst the normal physiological functions of the ACE2 system are protection against heart failure, myocardial infarction and hypertension. This can explain heart-related COVID-19 complications. Furthermore, defects in the ACE2 system were associated with lung disease, diabetes mellitus and gut dysfunction^(199)^. ACE2 is a Zn-metalloenzyme, and its normal function is therefore Zn-dependent. Thus, a likely explanation for the association of pre-existing Zn deficiency with COVID-19 complications is the decreased ACE2 activity reported for animals fed on a low-Zn diet^(201,202)^. ACE activity was even suggested as a biomarker for moderate Zn deficiency in patients with idiopathic taste impairment^(203)^. A Zn deficiency-related, mildly restricted ACE2 activity might not result in clinical symptoms. However, if ACE2 activity is further impaired by the virus, it might fall below a certain threshold and cause vascular complications, heart problems, gut disturbances and so on. Conversely, one study reported increased ACE2 activity in the lung tissue of Zn-deficient rats^(204)^. Thus, further clarification is needed before conclusions can be drawn regarding the relation to COVID-19. As Zn is a structural element of ACE2, the receptor’s conformation and subsequent affinity for the virus might be altered in patients with pre-existing Zn deficiency, which remains to be tested^(201)^. Furthermore, Zn deficiency might impair ACE2 expression, as was reported for other Zn-containing metalloenzymes^(182)^. Zn supplementation led to decreased Sirtuin-1 activity as found in cell culture. Interestingly, Zn removal from the closely related *Plasmodium falciparum* Sirtuin-2 deacetylase, resulted in structural collapse and malfunction of the enzyme. Since Sirtuin-1 is involved in regulating ACE2 transcription, this might result in disturbed ACE2 expression in patients with a Zn imbalance^(100,205,206)^.

In summary, pre-existing Zn deficiency might alter ACE2 expression, structure and/or activity in a tissue-specific manner, which could affect viral entry and pre-dispose to virus-induced tissue damage, but more and detailed studies are necessary to verify those speculation.

Acute kidney injury is another complication that can cause high mortality in COVID-19 patients^(207)^. The total incidence of acute kidney injury in COVID-19 patients is estimated to be about 4–9 %, while in a retrospective study, it was demonstrated that the percentage of patients with complications can reach 37–78 %^(207,208)^. In addition to increased epithelial barrier permeability and the infection with the virus via ACE2, Zn deficiency was associated with renal insufficiency^(85)^. Although described for rats, severe Zn deficiency that was observed in parallel decreased the glomerular filtration rates and renal blood flow, while renal vascular resistance increased^(209)^. The resulting renal insufficiency might be a pre-requisite for acute kidney injury and kidney failure during COVID-19. This hypothesis is supported by the finding that the role of Zn in renal function seems to be more crucial in diseased animals than in healthy ones. Tubulointerstitial nephropathy and glomerular haemodynamics were, for example, aggravated in rats with pre-existing Zn deficiency that were suffering from unilateral ureteral obstruction. Zn deficiency further increased the disease-related high expression of endothelin-1 in the glomeruli of the obstructed kidneys^(210,211)^. Since during kidney diseases and dialysis, Zn loss is increased, Zn deficiency is self-perpetuating and a vicious circle develops causing more severe disease^(212)^.

Diarrhoea was reported as a consequence of COVID-19 in a high number of cases^(77)^. The association of Zn deficiency with intestinal alterations and a leaky gut are well described in clinical investigations and Zn supplementation studies, and there are excellent reviews focusing on the underlying mechanism^(213–215)^.

Infection routes of COVID-19 may not include the intestinal tract. However, the leaky gut increases the risk of secondary infections, and intestinal morbidities as commensals are able to enter the human body^(216,217)^, especially if the immune system is otherwise occupied by the response to SARS-CoV-2. During renal diseases, nutrients not only Zn but also other elements important for an effective immune response can be lost from the body together with fluids. Consequently, dehydration and deficiency of various minerals can be expected^(213,218)^.

Finally, it is not without reason that Zn supplementation, especially of children in developing countries, is recommended by the WHO to prevent and treat diarrhoea, underlining the relevance of Zn for preserving a healthy gut, as a basic step towards improving the overall health status of individuals^(219)^.

## Pre-existing Zn deficiency decreases wound healing and tissue regeneration

Long-term consequences of COVID-19 including the damage to multiple organs are becoming more and more apparent. This is of course partly due to the severe damage caused by the virus but also due to slow and inefficient recovery and healing. Again, there are striking parallels between COVID-19 symptoms and impaired healing observed in Zn deficiency^(220,221)^, as found during *ex vivo* investigation of differentiated human airway epithelium and described by several research groups^(150,154)^.

In Zn-deficient rats, intestinal cell proliferation and the quality of intestinal wound healing after intestinal surgery were decreased compared with Zn-adequate controls. This was explained by higher expression of matrix metalloproteinases 2, 9 and 13 and decreased expression of Ki67 (proliferation marker). In addition, the collagen type I:III ratio was reduced in the Zn-deficient animals^(222)^. Whereas collagen type III dominates the early phase of wound healing, collagen type I rather represents late phase wound healing.

When the influx of Zn into the liver after partial hepatectomy was inhibited in a knock-out mutant of the Zn importer Zip14 in mice, proliferation of hepatocytes was significantly decreased^(223)^. Pre-existing Zn deficiency had similar effects regarding regeneration of heart and lungs. Moreover, Zn supplementation improved the recovery from ischaemia as for example shown in rats where Zn was added during re-perfusion or to the diet^(154,224,225)^.

A Zn-adequate nutrition may thus also be relevant during recovery from COVID-19.

## Zinc deficiency as pre-requisite to virus-induced neuronal damage and loss in smell and taste

In healthy and Zn-adequate individuals, the brain is usually separated from most of the immune cells by the blood–brain barrier. If the blood–brain barrier is damaged, for example, due to high levels of matrix metalloproteinase-9 or other matrix-degrading factors, the brain can easily be infiltrated by immune cells as well as by pathogens, causing neuronal damage^(226)^. Thus, entry of a virus into the brain and subsequent damage of the neuronal system culminating in disturbances of their sensory function might be expected during severe Zn deficiency.

Neurological complications of COVID-19 include meningitis and encephalitis, followed by delirium and coma, acute disseminated encephalomyelitis, myelitis, Guillain-Barré syndrome and cerebrovascular complications (stroke, transient ischaemic attack, central nervous system vasculitis)^(227)^. However, in comparison with patients with respiratory complications, the proportion of patients with neurological manifestations of COVID-19 might be rather small. Since a high percentage of the world’s population is likely to be infected with the virus, the total number of patients with neuronal complication might be expected to be high. Moreover, lifelong disabilities can result from encephalitis and stroke. Psychosis and paralysis are also discussed as COVID-19-related^(227,228)^. Subsequent health, social, care and economic costs to society will be high^(227,228)^. Although the exact mechanisms underlying the neurological disturbances in COVID-19 patients are so far not clearly defined, a combination of direct viral invasion with secondary effects of the over-responding immune system is likely.

Serum Zn deficiency has been related to neuronal conditions such as autism, depression, psychosis, Alzheimer’s disease, stroke and schizophrenia. Disturbed neurogenesis and elevated apoptosis of neuronal cells, which can result in defects in learning and memory, were described during Zn deficiency, as was shown in animals fed on a Zn-deficient diet. Retrospective studies on stroke patients also suggest a clinical significance for serum Zn deficiency^(229–235)^. The increase in neuronal apoptosis might involve mitochondrial p53 as well as p53-dependent caspase-mediated mechanisms as shown *in vitro* ^(236)^. Moreover, a deficiency in synaptic Zn, achieved by Zn chelation, elevated the susceptibility to epileptic seizures in rodents^(237,238)^. Also, Zn deficiency reduces the amount of Zn available for signal transmission and processing of information, considering that Zn functions as a neurotransmitter, as reviewed in detail elsewhere^(239)^. Zn is usually packaged into synaptic vesicles of a large sub-population of excitatory neurons for the purpose of neurotransmission. In addition, Zn functions as an important neuromodulator in the olfactory bulbs in rodents^(240,241)^. Restricting the release of Zn by knocking out the Zn exporter ZnT3 inhibited cell proliferation and neuronal differentiation in the adult hippocampus in mice^(242)^. Surprisingly, Zn in the brain remains unaltered or might even be elevated and involved in Alzheimer-related plaque formation in Zn-deficient animals and humans^(243–245)^. Thus, the relevance of direct effects of Zn deficiency to explain neuronal damage and defects in brain function awaits further data to assist verification. However, ROS, reactive nitrogen species and matrix metalloproteinase-9, which can cross the blood–brain barrier, are elevated during Zn deficiency and affect blood–brain barrier integrity, thus explaining the neuronal damage that has been found *in vitro* and *in vivo* ^(246,247)^.

Although not in itself life threatening, descriptions of disturbed sense of smell or taste, or both, in COVID-19 patients have accumulated^(248–251)^. An association between Zn deficiency and the (partial) loss of smell, taste or both has been described in several studies^(252,253)^. However, underlying mechanisms are so far not clear. Thus, a connection between Zn deficiency and the disturbances in taste and smell in COVID-19 patients must be carefully analysed in future studies. Extrapolating from the literature, however, still suggests some logical associations.

## The elderly: a risk group not only for Zn deficiency

The above-discussed consequences of Zn deficiency are relevant for all age groups. However, in a large number of subjects older than 65 years, co-morbidities may exist. Thus, the association of the age-related decline of serum Zn with the high susceptibility of the elderly for severe COVID-19 is hard to estimate. Instead, we would like to point out that although this article’s focus is Zn, a deficiency in other nutritional elements could also worsen COVID-19 prognosis^(3,254,255)^. Especially, the elderly suffer not only from Zn deficiency but often from inadequate nutrition. Thus, their nutritional status should generally be checked regularly. It was estimated that the prevalence of inappropriate nutrition risk in Europe is 8·5 % in the community setting, 17·5 % in residential care and 28 % in hospitalisation for individuals ≥65 years^(256)^. The evidence of the relationship between inappropriate nutrition, immunosenescence and the higher morbidity and mortality from COVID-19 in elderly patients was recently discussed^(3,254)^. Those articles may be consulted in regard to options especially for supporting the aged population in addition to Zn supplementation. The articles provide an elaboration on the impact of malnutrition on the immune system specifically of older subjects including cell-mediated immunity, cytokine production and phagocytic function^(3,6,7,254)^.

However, we believe that Zn supplementation of groups at risk of Zn deficiency and especially in case of the elderly can significantly reduce the severity of infectious diseases such as COVID-19, especially when combined with a generally optimised and nutritious diet, and physical exercise^(6,7,254)^.

## Next step: clinical trials

Based on the available literature, this article suggests a multitude of mechanisms as to how pre-existing Zn deficiency poses a risk of higher susceptibility to SARS-CoV-2 infections and a more severe progression of disease. To test this hypothesis, clinical studies are necessary and some are already registered^(257)^ (online Supplementary Table S1). Moreover, first clinical data support the hypothesis that serum Zn levels are decreased in COVID-19 patients and that disease severity and mortality might be inversely correlated with serum Zn concentration^(10,258)^. A decreased serum Zn level might perhaps be expected in COVID-19 patients due to the strong inflammatory response. Indeed, in serum samples from thirty-five patients with COVID-19, Zn levels were below those from healthy controls^(10)^. Furthermore, in a study of pregnant women, COVID-19 was associated with lower serum Zn levels and serum Zn was negatively correlated with inflammatory markers^(259)^. Thus, subjects starting out with an inherent Zn deficiency might be expected to be less well prepared for a COVID-19-induced decrease in serum Zn. In this regard, serum samples from non-survivors of COVID-19, taken at various time points, showed that the majority were below the threshold categorised as Zn-deficient. This was also noted for half of the surviving patients. The same study also found a Se deficiency in the majority of patients. The levels of Selenoprotein P and Zn in relation to the age of the subject were identified as reliable prognostic indicators for COVID-19 survival^(10)^. Analysis and correction of Se and Zn status were recommended. Another study also found that a significant number of COVID-19 patients were Zn deficient. Here, Zn-deficient patients revealed more complications, a prolonged hospital stay and higher mortality^(258)^. Low Zn levels in COVID-19 patients at clinical admission were associated with poor disease outcomes^(198)^. Finally, in Sakai City Medical Center (Osaka, Japan), most severely ill patients with COVID-19 showed Zn deficiency. Regarding those patients, critical illness could be predicted by serum Zn values. The authors thus suggest serum Zn levels as a predictive factor for a critical illness of COVID-19^(260)^. Additional studies on correlating serum Zn levels with disease severity are ongoing^(261)^.

The data we present strongly suggest that individuals with severe pre-existing Zn deficiency should be included in potential risk groups for COVID-19. We also suggest that prophylactic Zn supplementation, addressing mild pre-existing Zn deficiency, would be more promising than using Zn for the treatment of active disease. In several registered studies, Zn supplementation of groups with high risk of close contact with SARS-CoV-2, including medical or military personnel, is being investigated. Finally, the use of Zn supplementation alone or in combination with other treatment strategies is being tested in clinical studies. First data on the benefits of Zn supplementation as monitored by improved disease status in four confirmed cases of COVID-19 which were supplemented with up to 200 mg of elemental Zn per d were recently published^(262)^. However, only a minimal effect of Zn on the survival of Zn treated (100 mg elemental Zn per day) *v*. untreated COVID-19 patients was found by others^(263)^. Supplementation studies using Zn together with the ionophore chloroquine have so far produced contradictory results^(264–268)^.

Combining the Zn-related data from descriptive, preventive and treatment studies will be necessary to increase our knowledge of the importance of Zn homoeostasis during COVID-19 infections and for developing optimal Zn-based supplementation strategies.

## Conclusion

Zn is not without reason called an ‘essential’ trace element. Although its single actions on the various cells of the human body might be small and the symptoms of mild to moderate Zn deficiency are rather subtle, the pre-existing lack of Zn in combination with a pathogen such as SARS-CoV-2 can be detrimental and life threatening. Unfortunately, the current data for COVID-19 patients do not allow to distinguish, whether the low serum Zn levels repeatedly found are elicited by virus-induced inflammation, or are reflecting a pre-existing Zn deficiency which cause a more severe disease. Irrespective of this ambiguity, it is quite obvious that groups at risk of Zn deficiency may also be at risk of severe progression of COVID-19, in which the literature on the effects of Zn deficiency, summarised in this article, emphasises. Still, this hypothesis needs to be tested experimentally in clinical studies, some of which are currently in progress. At present, the hypothesis is only supported by data derived largely from animal and cell culture models of Zn deficiency.

This article underlines the various ways as to how a vicious circle of pre-existing, low-grade Zn deficiency and mild pathogen-induced symptoms, followed by increased loss of Zn from the body and the switch to more severe symptoms and serious complications, can be generated. Especially since Zn and its deficiency can have a wide variety of individually very different effects, the consequences of pre-existing Zn deficiency in combination with a pathogen like SARS-CoV-2 that causes so many different symptoms and complications by itself are almost impossible to predict. However, as a conclusion, it can be assumed that Zn deficiency represents a risk for severe progression of SARS-CoV-2-induced disease and a high mortality therefrom. As Zn supplementation is cost-effective and can be regarded as safe, it is highly recommended to supplement individuals who are at risk of Zn deficiency. Finally, we would like to add that more attention should be paid to monitoring nutritional status, since minerals and trace elements are inevitably associated with an efficient immune response. Collaborations between the wide range of clinical and research expertise from the nutritional field along with those involved in intensive care treatment, forming a COVID-19 Nutrition Network is desirable.

## Supplementary material

For supplementary material accompanying this paper visit https://doi.org/10.1017/S0007114521000738. click here to view supplementary material
