# Supplementary material for: Zinc deficiency as a possible risk factor for increased susceptibility and severe progression of Corona Virus Disease 19
Source: Br J Nutr. 2021 Mar 1;127(2):214–32. doi: 10.1017/S0007114521000738 (PMC8047403; doi:10.1017/S0007114521000738)
Supplement: Supplementary file 1 [file S0007114521000738sup001.docx]

**Supplementary Table 1: Registered Clinical Trials**

Currently registered clinical studies are summarized (last updated December 3^rd^, 2020). In observational studies, zinc is measured in samples from COVID-19 patients without further intervention. In preventive studies, healthy volunteers, where no SARS-CoV-2 was observed, were supplemented with zinc, while in the treatment studies zinc alone or in combination with other therapeutic strategies, was given to patients with confirmed COVID-19.

To facilitate comparison between the different studies, the elemental zinc concentration that was given per day was calculated where possible and this data included in the Table. Moreover, the total concentration given per day is indicated as well as the total duration of the respective intervention, rather than the actual dosage regime. For intake recommendations and study details (twice a day, before meals, etc), please refer to the individual study details listed in the registry homepages.

AU: Australia; BD: Bangladesh; BR: Brazil; c.n.s.: compound not specified; d: day; DHA; Docosahexaenoic acid; DK: Denmark; DZ: Algeria, EG: Egypt; EPA: eicosapentaenoic acid; ET: Ethiopia; FR: France; GB: United Kingdom; GLA: gamma-linolenic acid; HCQ: hydroxychloroquine; IN: India; IR: Iran; IU: international units; i.v.: intra venous; LMWH: low molecular weight heparin; mo: month; MX: Mexico; n.i. not indicated (zinc compound and concentration); ONS: oral nutrition supplement; PK: Pakistan; Ref: references; req: required; SA: Saudi Arabia; SG: Singapore; TN: Tunisia; TR: Turkey; USA: United States of America; Vit.: vitamin; wk: week(s); y: years; ZnAc: zinc acetate; ZnCitr: zinc citrate; ZnCl_2_ : zinc chloride; ZnGlc: zinc gluconate; ZnSO_4_: zinc sulphate

| Trial ID & Title | country | Status | Population | Strategy / Intervention | Elemental zinc [mg/d] | Ref |
| --- | --- | --- | --- | --- | --- | --- |
| OBSERVATORY | | | | |  |  |
| NCT04407572, Evaluation of the Relationship Between Zinc Vitamin D and b12 Levels in the Covid-19 Positive Pregnant Women [sic] | TR | complete | 18 – 45y pregnant females | Analysis of serum zinc, Vit.D, Vit.B_12_ | - | ^(1)^ |
| NCT04584424, Prognostic Factors and Outcomes of COVID-19 Cases in Ethiopia: Multi-Site Cohort Study. | ET | not yet recruiting | Child, Adult, older Adult | Analysis of prevalence of inadequate nutrient intake, including zinc  Analysis of relevance of nutritional deficiency measured in serum, including zinc |  | ^(2)^ |
| PREVENTIVE | | | | |  |  |
| EudraCT 2020-001363-85, COVID-19 Prophylaxis with hydroxychloroquine, Vitamin D, and Zinc supplementation in Danish nursing home residents – a randomized controlled trial | DK | prema-turely ended | Healthy nursing home residents ≥ 65y | 1) HCQ, Vit.D, zinc  2) control: no intervention  both 90 d | n.i. | ^(3)^ |
| NCT04377646, A Study of Hydroxychloro-quine and Zinc in the Prevention of COVID-19 Infection in Military Healthcare Workers | TN | not yet recruiting | 18 – 65y | 1) initial: HCQ 400 mg/d, for 2d,  then: HCQ 400 mg/wk, zinc 15 mg/d, total 2 mo 2) initial: HCQ 400 mg/d, for 2d,  then: HCQ 400 mg/wk, placebo, total 2 mo  3) placebo total 2 mo | 15  c.n.s. | ^(4)^ |
| NCT04584567, Covid-19 Infection Prophylaxis With Low Dose of Doxycycline and Zinc in Military Health Care Workers. | TN | not yet recruiting | 18 – 65y  healthy probands at risk | 1) doxycycline 100 mg/d, zinc 15 mg/d  2) doxycycline 100 mg/d, placebo  3) placebo | 15  c.n.s. | ^(5)^ |
| NCT04335084, A Randomized, Double-Blind, Placebo-Controlled Phase IIa Study of Hydroxychloroquine, Vitamin C, Vitamin D, and Zinc for the Prevention of COVID-19 Infection | US | recruiting | ≥ 18y, healthy, medical personnel | 1) initial: HCQ, 1d, then: Vit.C, Vit.D, zinc, 12 wk  2) Vit.C, Vit.D, zinc, 12 wk | n.i. | ^(6)^ |
| NCT04446104, A Randomized Open-label Prophylaxis Trial Among Migrant Workers at High-risk of Covid-19 (DORM Trial) | SG | complete | 21 – 60y, healthy male in dormatory | 1) initial: HCQ 400 mg/d, 1d, then: 200 mg/d,42d 2) zinc 80 mg/d + Vit.C 500 mg/d, 42d  3) Povidoneiodine throat spray, 42d  4) Ivermectin 12 mg/d, 1d  5) control: Vit.C 500 mg/d, 42d | 80  c.n.s. | ^(7)^ |
| NCT04384458, COVID-19 Prophylaxis With Hydroxychloroquine Associated With Zinc For High-Risk Healthcare Workers Involved In Suspected, or Confirmed Cases of COVID-19. | BR | not yet recruiting | 18 – 70y, healthcare workers, COVID-19 patient contact | 1) initial: HCQ 800 mg/d, 1d, then 400 mg/day 4d then: 400 mg/every 5d, 7 wk  + ZnSO_4_ 66 mg/d, 50 d  2) control: no intervention | 15 | ^(8)^ |
| NCT04326725, Proflaxis for Healthcare Professionals Using Hydroxychloroquine Plus Vitamin Combining Vitamins C, D and Zinc During COVID-19 Pandemia: An Observational Study [sic] | TR | active, not recruiting | 20 – 90y  healthcare professio-nals & 1^st^ degree relatives | HCQ: 200 mg/ every 3 wk + daily Vit. C  + daily zinc | n.i. | ^(9)^ |
| NCT04551339, Zinc Versus Multivitamin Micronutrient Supplementation to Support Immune Health in the Setting of COVID-19 Pandemic: A Randomized Study. | US | enrolling by invitation | 18 – 90y without COVID-19 infection | 1) ZnOx 69,6 mg/d (elemental), Cu 1.6 mg/d, Vit.A 8.593 mcg/d, Vit.C 452 mg/d, Vit.E 180 (PreserVision AREDS), 3 mo  2) Multivitamin supplementation (Centrum adult)  3 mo | 69.6 | ^(10)^ |
| TREATMENT | | | | |  |  |
| NCT04412746, Prevalence of Diabetes Among Hospitalized Patients With Covid-19 in West of Algeria. Identification of Diabetes-related Associated Factors Severe Forms | DZ | recruiting | ≥ 16y, diabetes & confirmedCOVID-19 | 1) HCQ: 600 mg/d, 10 days  + Azithromycin 500 mg/d, 1d; then 250 mg/d, 4d + ZnSO_4_ 220 mg/d, 5 d + LMWH + Metformin (no Insulin)  2) Lopinavir 800 mg/d, ritonavir 200 mg/d, 5 -7d | 50 | ^(11)^ |
| NCT04621149, A Phase 2 Screening Study of Candidate Non-prescription Treatments for COVID-19: A Patient-driven, Randomized, Factorial Study Evaluating Patient-reported Outcomes (PROFACT-01). | n.i. | not yet recruiting | 20 – 70y  non-hospita-lized | 1) Placebo  2) ZnAc  3) Chlorine dioxide aqueous solution (AS)  4) ZnAc + “AS”  5) Famotidine, lactoferrin and green tea extract  6) Green tea extract + “AS”  7) ZnAc + green tea extract  8) ZnAc + green tea extract + “AS”  7d | n.i. | ^(12)^ |
| NCT04507867, Effect of a Nutritional Support System to Reduce Complications in Patients With Covid-19 and Comorbidities in Stage III. | MX | not yet recruiting | 30 – 75y  confirmed COVID-19 need of oxygen | 1) Control 25d / until discharge  2) zinc 20 mg, part of Nutritional support system (NSS) up to 25d / until discharge | c.n.s. | ^(13)^ |
| NCT04621461, A Randomized, Placebo-Controlled Study Evaluating the Safety and Efficacy of Hydroxychloroquine, Azithromycin and Zinc for the Treatment of COVID-19 in the Outpatient Setting. | US | not yet recruiting | 30 years and older  outpatient setting | 1) HCQ 800 mg/d, 1d, then 400 mg/d 4d, Azithromycin 500 mg/d 1d, then 250 mg/d, 4d, ZnSO_4_ 220 mg/d  2) same as 1), no zinc  Study duration 30d | 50 | ^(14)^ |
| NCT04641195, A Randomized Trial to Determine the Effect of Vitamin D and Zinc Supplementation for Improving Treatment Outcomes Among COVID-19 Patients in India. | IN | Not yet recruiting | ≥18y confirmedCOVID-19 | 1) ZnGlc 40 mg/d (elemental), Vit.D3 180.000IE 1d, then 2000 IE/d, 8 wks  2) ZnGlc 40 mg/d elemental), placebo  3) Vit.D3 180.000IE 1d, then 2000 IE/d, placebo  4) placebo | 40 | ^(15)^ |
| NCT04558424, Randomized, Double -Blind, Placebo Controlled, Trial to Evaluate the Effect of Zinc and Ascorbic Acid Supplementation in COVID-19 Positive Hospitalized Patients in BSMMU. | BD | not yet recruiting | ≥18y confirmedCOVID-19  moderate to severe disease | 1) ZnGlc 220 mg/d, Vit.C 1 mg/d, 10d  2) placebo, 10d | 32,2 | ^(16)^ |
| CTRI/2020/07/026340. Prospective study to assess therapeutic role of Zinc in COVID-19 patients | IN | not yet recruiting | 18–80y, COVID-19 patients | 1) ZnSO_4_ 100 mg/d + standard treatment, 14d  2) standard treatment, 14d | 22.5 | ^(17)^ |
| NCT04323228, Anti-inflammatory/Antioxi-dant Oral Nutrition Supplementation on the Cytokine Storm and Progression of COVID-19: A Randomized Controlled Trial. | SA | recruiting | 18 – 65y confirmedCOVID-19 | 1) daily ONS: protein 14.8 g, fat 22.2 g, carbohydrate 25 g, EPA 1.1 g, DHA 450 mg, GLA 950 mg, Vit.A 2840 IU, Vit.C 205 mg, Vit.E 75 IU, Selenium 18 µg, zinc 5.7 mg, up to 3 mo  2) daily iso-caloric-isonitrogenous supplement | 5.7 | ^(18)^ |
| NCT04334512, A Randomized, Double-Blind, Placebo-Controlled Phase IIa Study of Quintuple Therapy to Treat COVID-19 Infection. | US | recruiting | ≥ 18y  confirmedCOVID-19 | 1) HCQ, Azithromycin, Vit.C, Vit.D, zinc, 10d  2) placebo, Vit.C, VitD, zinc, 10d | n.i. | ^(19)^ |
| NCT04342728, Coronavirus Disease 2019 - Using Ascorbic Acid and Zinc Supplementation (COVIDAtoZ) Research Study A Randomized, Open Label Single Center Study. | US | enrolling by invitation | ≥18y confirmedCOVID-19 | 1) Vit.C 8000 mg/d  2) ZnGlc 50 mg /d  3) ascorbic acid 8000 mg/d + ZnGlc 50 mg/d  4) standard care  all 10d | 7.18 | ^(20)^ |
| NCT04373733, A Randomised Controlled Trial of Early Intervention in Patients HospItalised With COVID-19: Favipiravir and StaNdard Care vErsEs Standard CaRe [sic] | GB | recruiting | ≥ 18y  suspected/confirmedCOVID-19 | 1) Favipiravir: 3600 mg/d, 1d1, 1600 mg/d, 9d  2) HCQ: 800 mg/d, 1d, 400 mg/d, 9d + Azithromycin: 250 mg/d, 3d + ZnSO_4_: 250 mg/d, 10d  3) standard care | 57 | ^(21)^ |
| NCT04351490, Impact of Zinc and Vitamin D3 Supplementation on the Survival of Aged Patients Infected With COVID-19 | FR | not yet  recruiting | ≥ 60y  confirmedCOVID-19 | 1) ZnGlc 30 mg/d (elemental), VitD_3_ 2000 IU/d  2) no interventiom  both 2 mo | 30 | ^(22)^ |
| NCT04447534, Does Zinc Supplementation Enhance the Clinical Efficacy of Chloroquine/Hydroxychloroquine in Treatment of COVID-19? | EG | recruiting | ≥ 18y  confirmedCOVID-19 | 1) HCQ, zinc  2) HCQ | n.i. | ^(23)^ |
| ACTRN12620000454976, High-dose intravenous zinc (HDIVZn) as adjunctive therapy in COVID-19 positive critically ill patients: A pilot randomized controlled trial | AU | recruiting | ≥ 18y  confirmedCOVID-19 | 1) ZnCl_2_, i.v. 0.5 mg/kg/d, 7d  2) saline placebo, 7d | 0.5mg/  kg/d | ^(24)^ |
| IRCT20180425039414N2, The effect of zinc on the treatment and clinical course of patients with SARS-CoV-2 (COVID-19) | IR | recruiting complete | ≥18y confirmedCOVID-19 | 1) HCQ 800 mg/d at day 1, 400 mg/d remaining hospitalization, ZnSO_4_ 440 mg/d  2) HCQ 800 mg/d at day 1, 400 mg/d remaining hospitalization | 100 | ^(25)^ |
| IRCT20151228025732N52, A study on melatonin and Vitamin C and zinc efficacy in patients with COVID19 hospitalized in intensive care unit of Semnan Kowsar Hospital | IR | recruiting complete | confirmedCOVID-19 | 1) melatonin 40 mg/d, ascorbic acid 2 g/d, ZnSO_4_ 880 mg/d, 5d  2) standard care | 200 | ^(26)^ |
| NCT04472585, Efficacy of Subcutaneous Ivermectin With or Without Zinc and Nigella Sativa in COVID-19 Patients (SINZ-COVID-PK) | PK | recruiting | ≥18y confirmedCOVID-19  BMI 18-28 kg/m | 1) Ivermectin 200 µg/kg every 48 h  2) Ivermectin 200 µg/kg every 48 h, Nigella Sativa 80 mg/kg/day  3) Ivermectin 200 µg/kg every 48 h, ZnSO_4_ 60 mg/d  4) Placebo | 13.6 | ^(27)^ |
| HOMEOPATHIC STUDIES | | | | |  |  |
| CTRI/2020/06/026045, Evaluating the immune boosting ability of a homoeopathic therapeutic strategy involving a nosode Tuberculinum 1M, followed by Zincum Metallicum 6C, Chininum Arsenicosum 6C and Calc Phos 6x in asymptomatic novel corona virus disease (Covid-19 illness) vulnerable risk group | IN | not yet recruiting | 14 – 60y  no symptoms  at risk | 1) Tuberculinum 1M, 4 pills, Zincum Metallicum 6C, Chininum Arsenicosum 6C and Calc phos 6X twice daily for 14 days  2) no intervention | n.i. | ^(28)^ |
| CTRI/2020/06/025998, To Determine the Efficacy of An Ayurvedic Preparation Raj Nirwan Bati (RNB) on symptomatic COVID-19 Patients: A Double-Blind Randomized Controlled Trial | IN | Not yet recruiting | 18 – 99y confirmedCOVID-19 | 1) Raj Nirwan Bati capsule (250 mg/day): mercury, sulphur, gold, silver, clamina perpeta, arsenic trioxide, black pepper, naag damanti, celery, zinc, niramish  2) placebo, both 12d | n.i. | ^(29)^ |
| CTRI/2020/06/025874, A randomized open labeled clinical study to compare the effectiveness of Kabasura kudineer and Vitamin-C Zinc supplementation in the management of asymptomatic SARS-CoV-2 patients | IN | Not yet recruiting | confirmed COVID-19 without symptoms | 1) Kabasura kudineer: 60 - 120 ml/d  2) Zinc 200 mg/d, Vit.C 120000 IU/d  both 14d | c.n.s. | ^(30)^ |
| CTRI/2020/06/025856, An open clinical evaluation of selected siddha regimen in expediting the management of covid 19 - A randomized controlled study | IN | Not yet recruiting | 18 -60y  confirmed COVID-19 | 1) Kabasura kudineer 120 ml/d, Vasantha kusumakaram Mathirai 2 tablets/d, Thippili Rasayanam 4 g/d, Adathodai Manapagu 30 ml/d  2) HCQ 800 mg/d, 1d, then 400 mg/d, Azithromycin 500 mg/d, 5d, Omeprazole 40 mg/d, Vit.C 2 units/d, Zn 2 units/d, both 14d | n.i. | ^(31)^ |
| CTRI/2020/05/025215, A prospective, single centre, randomized open labelled comparative clinical study to evaluate the effectiveness of Siddha medicine, Kabasura kudineer and vitamin c-zinc supplemen-tation in the management of asymptomatic COVID 19 patients. | IN | complete | 18 – 55y  confirmedCOVID-19, no symptoms | 1) Kabasura Kudineer 120 ml/d  2) Vit.C 60000 IU/d, + Zinc 100 mg/d  both 14d | c.n.s. | ^(32)^ |
| OTHER STUDIES INCLUDING ZINC COMPOUNDS | | | | | | |
| NCT04395768, Therapies to Prevent Progression of COVID-19, Including Hydroxychloroquine, Azithromycin, Zinc, Vitamin D, Vitamin B12 With or Without Vitamin C, a Multi-centre, International, Randomized Trial: The International ALLIANCE Study. | AU | recruiting | ≥ 18y  active COVID-19 | 1) Inpatients: Vit.C, i.v. 200 mg/kg/d, 1d, 400 mg/kg/d, 7 days (max: 50g/d). + comparator  2) Outpatients: Vit.C, i.v. 200 mg/kg/d, 1d, 3g/d, 7d + comparator  3) Comparator: HCQ 800 mg/d, 1d, 400 mg/d, 6d; Azithromycin 500 mg/d, 1d, 250 mg/d, 4d ZnCitr 30 mg/d (elemental),  Vit.D_3_ 5,000 IU/d, 14 d, Vit.B_12_ 500 mg/d, 14d | 30 | ^(33)^ |
| NCT04370782, A Randomized Study Evaluating the Safety and Efficacy of Hydroxychloroquine and Zinc in Combination With Either Azithromycin or Doxycycline for the Treatment of COVID-19 in the Outpatient Setting. | US | recruiting | 30 - 59y or ≥ 60y  confirmedCOVID-19 | 1) HCQ 800 mg/d, 1d, 400 mg/d, 4d, ZnSO_4_ 220 mg/d, 5d, Azithromycin 500 mg/d,1d, 250 mg/d 4d  2) HCQ 800 mg/d, 1d, 400 mg/d, 4d, ZnSO_4_ 220 mg/d, 5d, Doxycycline 200 mg/d, 5d | 50 | .^(34)^ |
| NCT04468139, The Study of Quadruple Therapy Zinc, Quercetin, Bromelain and Vitamin C on the Clinical Outcomes of Patients Infected With COVID-19 | SA | recruiting | ≥ 18y confirmedCOVID-19 | Quercetin 500 mg/d, Bromelain 500 mg/d, Vit.C 1000 mg/d, zinc 50 mg/d, 5-10d or patient improves or discharged | 50 | ^(35)^ |
| NCT04491994, Clearing the Fog: Is Hydroxychloroquine Effective in Reducing COVID-19 Progression (COVID-19) | PK | completed | 18 – 80y confirmedCOVID-19 | 1) HCQ 800 mg, 1d, 400 mg/d, 5d, Vit C 2g/d, Vit.D 1µg/d, Zinc 50 mg/d, paracetamol as req.  2) Vit C 2g/d, Vit D 1µg/d, Zinc 50 mg/d, paracetamol as req. | 50 | ^(36,37)^ |
| NCT04542993, Can SARS-CoV-2 Viral Shedding in COVID-19 Disease be Reduced by Resveratrol-assisted Zinc Ingestion, a Direct Inhibitor of SARS-CoV-2-RNA Polymerase? A Single Blinded Phase II Protocol (Reszinate Trial). | SE | recruiting | 18 – 75y confirmed  COVID-19 | 1) zinc 150 mg/d (elemental), resveratrol 4 g/d, 5d  2) placebo, 5d | 150 | ^(38)^ |
| NCT04482686, A Phase II Double-Blind Randomized Placebo-Controlled Trial of Combination Therapy to Treat COVID-19 Infection**.** | US | not yet recruiting | 18 – 78y  confirmed COVID-10 | 1) ZnSO_4,_ Ivermectin, Doxycycline, Vit.D3, Vit.C, 10 d  2) ZnSO_4,_ Vit.D3, Vit.C, 10 d | n.i. | ^(39)^ |

References

1. ClinicalTrials.gov Evaluation of the Relationship Between Zinc Vitamin D and b12 Levels in the Covid-19 Positive Pregnant Women. https://ClinicalTrials.gov/show/NCT04407572.

2. ClinicalTrials.gov Prognostic Factors and Outcomes of COVID-19 Cases in Ethiopia. https://ClinicalTrials.gov/show/NCT04584424.

3. clinicaltrialsregister.eu (2020) COVID-19 Prophylaxis with hydroxychloroquine, Vitamin D, and Zinc supplementation in Danish nursing home residents – a randomized controlled trial: Medication for prevention of Corona-virus for Danish nursing home residents. COVID-19 PREVENTION. https://www.clinicaltrialsregister.eu/ctr-search/trial/2020-001363-85/DK#P.

4. ClinicalTrials.gov A Study of Hydroxychloroquine and Zinc in the Prevention of COVID-19 Infection in Military Healthcare Workers. https://ClinicalTrials.gov/show/NCT04377646.

5. ClinicalTrials.gov OD-doxy-PNVCOV Old Drug " DOXY " for Prevention of New Virus Virus OD-doxy-PNV. https://ClinicalTrials.gov/show/NCT04584567.

6. ClinicalTrials.gov A Study of Hydroxychloroquine, Vitamin C, Vitamin D, and Zinc for the Prevention of COVID-19 Infection. https://ClinicalTrials.gov/show/NCT04335084.

7. ClinicalTrials.gov A Preventive Treatment for Migrant Workers at High-risk of Covid-19. https://ClinicalTrials.gov/show/NCT04446104.

8. ClinicalTrials.gov COVID-19 Prophylaxis With Hydroxychloroquine Associated With Zinc For High-Risk Healthcare Workers. https://ClinicalTrials.gov/show/NCT04384458.

9. ClinicalTrials.gov Proflaxis Using Hydroxychloroquine Plus Vitamins-Zinc During COVID-19 Pandemia. https://ClinicalTrials.gov/show/NCT04326725.

10. ClinicalTrials.gov Zinc Versus Multivitamin Micronutrient Supplementation in the Setting of COVID-19. https://ClinicalTrials.gov/show/NCT04551339.

11. ClinicalTrials.gov Covid-19 and Diabetes in West of Algeria. https://ClinicalTrials.gov/show/NCT04412746.

12. ClinicalTrials.gov An Outpatient Study Investigating Non-prescription Treatments for COVID-19. https://ClinicalTrials.gov/show/NCT04621149.

13. ClinicalTrials.gov Effect of a Nss to Reduce Complications in Patients With Covid-19 and Comorbidities in Stage III. https://ClinicalTrials.gov/show/NCT04507867.

14. ClinicalTrials.gov Hydroxychloroquine, Azithromycin and Zinc for the Treatment of COVID-19 in the Outpatient Setting. https://ClinicalTrials.gov/show/NCT04621461.

15. ClinicalTrials.gov Vitamin D and Zinc Supplementation for Improving Treatment Outcomes Among COVID-19 Patients in India. https://ClinicalTrials.gov/show/NCT04641195.

16. ClinicalTrials.gov RCT,Double Blind, Placebo to Evaluate the Effect of Zinc and Ascorbic Acid Supplementation in COVID-19 Positive Hospitalized Patients in BSMMU. https://ClinicalTrials.gov/show/NCT04558424.

17. ctri.nic.in/ (2020) Prospective study to assess therapeutic role of Zinc in COVID-19 patients: To study the role of Zinc combined with standard treatment for COVID-19. http://www.ctri.nic.in/Clinicaltrials/pmaindet2.php?trialid=44929 (accessed July 2020).

18. ClinicalTrials.gov Anti-inflammatory/Antioxidant Oral Nutrition Supplementation in COVID-19. https://ClinicalTrials.gov/show/NCT04323228.

19. ClinicalTrials.gov A Study of Quintuple Therapy to Treat COVID-19 Infection. https://ClinicalTrials.gov/show/NCT04334512.

20. ClinicalTrials.gov Coronavirus 2019 (COVID-19)- Using Ascorbic Acid and Zinc Supplementation. https://ClinicalTrials.gov/show/NCT04342728.

21. ClinicalTrials.gov Early Intervention in COVID-19: Favipiravir Verses HydroxycholorquiNe & Azithromycin & Zinc vErsEs Standard CaRe. https://ClinicalTrials.gov/show/NCT04373733.

22. ClinicalTrials.gov Impact of Zinc and Vitamin D3 Supplementation on the Survival of Aged Patients Infected With COVID-19. https://ClinicalTrials.gov/show/NCT04351490.

23. ClinicalTrials.gov Zinc With Chloroquine/Hydroxychloroquine in Treatment of COVID-19. https://ClinicalTrials.gov/show/NCT04447534.

24. ANZCTR.org.au High-dose intravenous zinc (HDIVZn) as adjunctive therapy in COVID-19 positive critically ill patients: A pilot randomized controlled trial. https://www.anzctr.org.au/Trial/Registration/TrialReview.aspx?id=379579&isReview=true.

25. irct.ir (2020) The effect of zinc on the treatment and clinical course of patients with SARS-cov2 (COVID-19). https://www.irct.ir/trial/47516 (accessed July 2020).

26. irct.ir (2020) A study on melatonin and Vitamin C and zinc efficacy in patients with COVID19 hospitalized in intensive care unit of Semnan Kowsar Hospital. https://www.irct.ir/trial/46963 (accessed July 2020).

27. ClinicalTrials.gov Efficacy of Subcutaneous Ivermectin With or Without Zinc and Nigella Sativa in COVID-19 Patients. https://ClinicalTrials.gov/show/NCT04472585.

28. ctri.nic.in/ (2020) Evaluating the immune boosting ability of a homoeopathic therapeutic strategy involving a nosode Tuberculinum 1M, followed by Zincum Metallicum 6C, Chininum Arsenicosum 6C and Calc Phos 6x in asymptomatic novel corona virus disease (Covid-19 illness) vulnerable risk group. http://ctri.nic.in/Clinicaltrials/showallp.php?mid1=44677&EncHid=&userName=/2020/06/026045 (accessed July 2020).

29. ctri.nic.in/ (2020) To Determine the Efficacy of An Ayurvedic Preparation Raj Nirwan Bati (RNB) on symptomatic COVID-19 Patients: A Double-Blind Randomized Controlled Trial: Efficacy of An Ayurvedic Preparation Raj Nirwan Bati (RNB) on symptomatic COVID-19 Patient. http://ctri.nic.in/Clinicaltrials/showallp.php?mid1=44707&EncHid=&userName=2020/06/025998 (accessed July 2020).

30. ctri.nic.in/ (2020) A randomized open labeled clinical study to compare the effectiveness of Kabasura kudineer and Vitamin-C Zinc supplementation in the management of asymptomatic SARS-CoV-2 patients: To observe the effect of Siddha formulation Kabasura kudineer in COVID 19 patients. http://ctri.nic.in/Clinicaltrials/showallp.php?mid1=44684&EncHid=&userName=2020/06/025874 (accessed July 2020).

31. ctri.nic.in/ (2020) An open clincal evaluation of selected siddha regimen in expediting the management of covid 19 - A randomized controlled study: Evaluation of siddha regimen in the management of covid 19. http://ctri.nic.in/Clinicaltrials/showallp.php?mid1=44606&EncHid=&userName=2020/06/025856 (accessed July 2020).

32. ctri.nic.in/ (2020) A prospective, single centre, randomized open labelled comparative clinical study to evaluate the effectiveness of Siddha medicine, Kabasura kudineer and vitamin c-zinc supplementation in the management of asymptomatic COVID 19 patients.: Effectiveness of Siddha medicine, Kabasura kudineer and vitamin c-zinc supplementation in the management of Mild COVID 19 patients. http://ctri.nic.in/Clinicaltrials/showallp.php?mid1=43769&EncHid=&userName=2020/05/025215 (accessed July 2020).

33. ClinicalTrials.gov International ALLIANCE Study of Therapies to Prevent Progression of COVID-19. https://ClinicalTrials.gov/show/NCT04395768.

34. ClinicalTrials.gov Hydroxychloroquine and Zinc With Either Azithromycin or Doxycycline for Treatment of COVID-19 in Outpatient Setting. https://ClinicalTrials.gov/show/NCT04370782.

35. ClinicalTrials.gov The Study of Quadruple Therapy Zinc, Quercetin, Bromelain and Vitamin C on the Clinical Outcomes of Patients Infected With COVID-19. https://ClinicalTrials.gov/show/NCT04468139.

36. ClinicalTrials.gov Clearing the Fog: Is Hydroxychloroquine Effective in Reducing COVID-19 Progression. https://ClinicalTrials.gov/show/NCT04491994.

37. Kamran SM, Mirza Z-e-H, Naseem A et al. (2020) Clearing the fog: Is Hydroxychloroquine effective in reducing Corona virus disease-2019 progression: A randomized controlled trial.

38. ClinicalTrials.gov Can SARS-CoV-2 Viral Load and COVID-19 Disease Severity be Reduced by Resveratrol-assisted Zinc Therapy. https://ClinicalTrials.gov/show/NCT04542993.

39. ClinicalTrials.gov Trial of Combination Therapy to Treat COVID-19 Infection. https://ClinicalTrials.gov/show/NCT04482686.
